# Infectious Bovine Respiratory Diseases in Adult Cattle: An Extensive Necropsic and Etiological Study

**DOI:** 10.3390/ani11082280

**Published:** 2021-08-02

**Authors:** Laëtitia Dorso, Maud Rouault, Claire Barbotin, Christophe Chartier, Sébastien Assié

**Affiliations:** 1 INRAE, Oniris, BIOEPAR, 44300 Nantes, France; maud.rouault@oniris-nantes.fr (M.R.); barbotin.claire@hotmail.fr (C.B.); christophe.chartier@oniris-nantes.fr (C.C.); sebastien.assie@oniris-nantes.fr (S.A.); 2 Pathology Unit for Large Animals, Oniris, La Chantrerie, 44307 Nantes, France; 3 Clinic for Ruminants, Oniris, La Chantrerie, 44307 Nantes, France

**Keywords:** adult cattle, necropsy, bovine respiratory diseases, thromboembolic pneumonia, *Mannheimia haemolytica*

## Abstract

**Simple Summary:**

Animal necropsy can accurately determine the cause of its death. However, studies based on large-scale necropsies of cattle are rare because they require special skills and equipment. This study places particular emphasis on fatal respiratory diseases in adult cattle. The objectives of this study were to assess the importance of respiratory diseases as a cause of death in adult cattle and to determine associated lesions as well as associated pathogens of infectious causes of respiratory diseases in adult cattle. This study showed that respiratory diseases are the second leading cause of death in adult cattle after digestive diseases. Among respiratory diseases, we noticed a strong predominance of infectious pulmonary lesions, mainly characterized by fibrinous, hemorrhagic and/or necrotic bronchopneumonia. These bronchopneumonia are mainly associated with the detection of a bacteria, *Mannheimia haemolytica*. This study suggests that *Mannheimia haemolytica* should be included in the differential diagnosis of BRD in adult cattle.

**Abstract:**

In young cattle, bovine respiratory disease (BRD) is a major cause of death and *Mannheimia haemolytica* is a frequent pathogen. Knowledge of fatal BRD in adult cattle is more limited. We assessed the importance of infectious BRD as a cause of death in adult cattle and determined the associated pathogens. We analyzed data from 737 adult cattle necropsies at the Pathology Unit for Large Animals at Oniris, Nantes, France over a 6 year period (2013–2019). Each carcass was subjected to a complete necropsy. Lungs showing macroscopic lesions were classified into three categories: infectious primary pulmonary (IPP) lesions, thromboembolic pneumonia (TEP) and others (aspiration pneumonia, verminous pneumonia, and local extension of an extra-pulmonary inflammatory process). Half of the lungs with IPP macroscopic lesions were sampled for histology and submitted for polymerase chain reaction. BRD was the second leading cause of death (15.7%) after digestive diseases (32.2%). A strong predominance of IPP lesions (42.3%) and TEP lesions (39.6%) was also demonstrated. In IPP macroscopic lesions, fibrinous, hemorrhagic and/or hecrotic (FHN) bronchopneumonia accounted for 77.6% of macroscopic lesions. *Mannheimia haemolytica* was significantly associated with FHN bronchopneumonia macroscopic lesions. This study suggests that *Mannheimia haemolytica* should be included in the differential diagnosis of BRD in adult cattle.

## 1. Introduction

Bovine respiratory disease (BRD) complex encompasses numerous pathogenic processes and etiologies. BRD includes disorders involving both the upper and lower respiratory tracts [1]. Among lower respiratory tract disorders, infectious BRD is due to airborne pulmonary infection by viruses and bacteria, including mycoplasma, but also to the hematogenous spread of infection (thromboembolic pneumonia) and to parasites (verminous pneumonia), while non-infectious BRD includes atypical interstitial pneumonia (acute pulmonary emphysema and edema and fog fever), extrinsic allergic alveolitis (bovine famer’s lung) and aspiration pneumonia [1]. Numerous studies have reported the frequent implication of *Mannheimia haemolytica* as a main etiological agent in fatal infectious BRD in young cattle [2,3,4,5,6,7]. In necropsied adult cattle, high proportions of cows with fatal pneumonia have also been reported [7,8,9,10]. However, the etiology of these fatal cases in adult cattle remains poorly documented. To the best of our knowledge, description of the etiological agents has only been made in one recent study showing the emergence of fatal *Mannheimia haemolytica* infections in necropsied adult dairy cattle in the Netherlands [7].

Exhaustive field necropsies of adult cattle are difficult to perform for a veterinarian, due to the large carcass size, availability of suitable equipment and biosecurity considerations. Similarly, veterinary diagnostic laboratories with a large animal necropsy room are scarce. In 2013, the Pathology Unit for Large Animals (PULA), dedicated to large animal necropsy, was created at the Oniris Veterinary Medicine School, Nantes, France. This unit collects dead cattle in West France, and an ECVP-diplomate pathologist performs necropsies. Veterinarians request necropsies for diagnostic purposes and farmers generally assume its cost. These necropsies are supposed to give valuable information about causes of death in adult cattle in West France by reflecting the true underlying pathology leading to death.

The objectives of this study were (i) to assess the importance of BRD as a cause of death in adult cattle submitted for necropsy in West France and (ii) to determine macroscopic and histological lesions as well as associated pathogens of infectious causes of BRD in adult cattle.

## 2. Materials and Methods

### 2.1. Selection of Cases and Data Collection

This study was conducted from January 2013 to December 2019, including all cattle over 2 years of age submitted for necropsy to the PULA of Oniris, Nantes, France. For each necropsy, the age in years ((2–3), (4–5), (>5)), cattle breed type (beef or dairy), year of death ((2013–2015), (2016–2017), (2018–2019)) and season of death (autumn/winter, spring/summer, corresponding respectively to the periods of October to March and April to September) were recorded.

### 2.2. Macroscopic Lesions

Each carcass was subjected to a complete standardized necropsy to determine the cause of death [11]. A single operator (LD, ECVP dipl.) described all macroscopic lesions. We defined eight categories based on the affected organ system [12] as the principal cause of death: (i) cardiovascular, (ii) digestive, (iii) musculoskeletal, (iv) reproductive, (v) respiratory and (vi) systemic disorders, (vii) other disorders, and (viii) undetermined. Respiratory disorders included upper and lower respiratory disorders. “Other disorders” included intoxication, dermatologic, metabolic, neurological and urinary disorders. The last category—“Undetermined”—included cases where the cause of death could not be macroscopically clearly identified [10]. Each necropsied animal was classified into one category. For each category, examples of diagnosis are detailed in Table 1.

In the presence of a grossly visible primary pulmonary lesion, a standard examination and tissue sampling protocol for respiratory disease diagnostic purposes was employed. Briefly, the trachea, lungs, and heart were removed; the lungs were palpated and serially incised; and the trachea, bronchi, and pulmonary arteries were incised longitudinally. All grossly visible lesions were described, and pulmonary lesions were mapped graphically on a diagram of the lungs, according to the percentage of the lung affected. The color, distribution, palpation (consistency), demarcation, size and associated lesions were reported on this diagram, as described by Murray et al. [6]. Associated lesions included edema, pleuritis, abscessation, emphysema, bronchiectasis, BALT (bronchus-associated lymphoid tissue) hyperplasia, necrosis, purulent exudate, mucoid exudate and atelectasis. Macroscopic lung lesions were then classified into three categories: (i) infectious primary pulmonary (IPP) lesions corresponding to airborne contamination, (ii) thromboembolic pneumonia (TEP) lesions corresponding to hematogenous spread, and (iii) others (aspiration pneumonia, verminous pneumonia, and local extension of an extra-pulmonary inflammatory process).

IPP macroscopic lesions were then classified into four patterns: (i) suppurative bronchopneumonia, (ii) fibrinous, hemorrhagic and/or necrotic (FHN) bronchopneumonia, (iii) broncho-interstitial pneumonia, and (iv) association of suppurative pneumonia and broncho-interstitial pneumonia, according to the classification described by Murray et al. [6].

### 2.3. Histology

Histology was performed in order to further describe IPP macroscopic lesions. For financial reasons, only half of the affected lungs were sampled at random for histology. The sections were fixed in 10% neutral buffered formalin, embedded in paraffin wax, cut with a microtome, and stained with hematoxylin and eosin. Histological diagnoses were performed by a single operator (LD, ECVP dipl.), and were based on five histological patterns (suppurative bronchopneumonia, FHN bronchopneumonia, broncho-interstitial pneumonia, interstitial pneumonia and the association of suppurative pneumonia and broncho-interstitial pneumonia), according to the classification described by Murray et al. [6].

### 2.4. PCR Analysis

IPP macroscopic lesions sampled for histology were also submitted for polymerase chain reaction (PCR) analysis for detection of *Pasteurella multocida* (*P. multocida*), *Mannheimia haemolytica* (*M. haemolytica*), *Histophilus somni* (*H. somni*), *Mycoplasma bovis* (*M. bovis*), bovine respiratory syncytial virus (BRSV), bovine parainfluenza virus type 3 (BPIV-3) and bovine coronavirus (BCoV). Each PCR was carried out using commercial PCR kits (Bio-T kit *Mycoplasma bovis*/*Histophilus somni*, Bio-T kit *Mannheimia haemolytica*/*Pasteurella multocida*, Bio-T kit Bovine coronavirus and Bio-T kit BRSV/BPIV-3 (Biosellal, Dardilly, France)), according to the manufacturer’s instructions. Lung samples (40–50 mg) were removed, finely chopped with a sterile disposable scalpel in a sterile Petri dish and transferred to a tube containing 200 μL of sterile 1× PBS (phosphate buffered saline) buffer. After vortexing and brief centrifugation, extraction of the nucleic acids was performed from 100 μL of resuspended dilacerated organs, following the protocol RNeasy Mini Kit (Qiagen, Manchester, United Kingdom). Each 20 µL reaction contained 15 µL of the mastermix and 5 µL of template nucleic acid. Amplification was carried out according to the manufacturer’s recommendations: 1 cycle at 50 °C for 20 min, 1 cycle at 95 °C for 5 min followed by 40 cycles of 15 sec at 95 °C and 30 sec at 60 °C. Commercial positive controls (Biosellal, Dardilly, France) and a negative control (PBS) were used in all amplification runs for each PCR.

### 2.5. Statistical Analysis

Open-source environment R version 3.5.1. (R Development Core Team, Vienna, Austria) was used to perform the descriptive statistical analysis and to obtain frequency distributions. Associations between pathological findings (macroscopic and histological patterns) and sampling data (age, cattle breed type, year and season) were tested using a chi-square test. When the numbers within categories were less than five, a Fisher’s exact test was used. Differences were considered statistically significant where *p* < 0.05.

## 3. Results

### 3.1. BRD as the Second Highest Cause of Death

Seven hundred and thirty-seven adult cattle, originating from 576 farms, were necropsied. Respiratory disorders, characterized by lower respiratory tract macroscopic lesions evocative of BRD, were the second leading cause of death (115/737, 15.1%) after digestive disorders (237/737, 32.2%). Among digestive disorders, intestinal disorders accounted for 54% (128/237), peritonitis for 27.8% (66/237) and abomasal disorders for 8.9% (21/237). Other disorders affecting the oral cavity, esophagus, rumen or liver accounted for 9.3% (22/237). One necropsied animal (0.1%; 1/737) had an upper respiratory disorder.

A rather large number of cattle died of undetermined causes (102/737, 13.8%): in these cases, the cause of death could not be clearly identified, either because the state of preservation did not enable a relevant necropsic diagnosis (59/102, 57.8%), or because no significant macroscopic lesion was observed (43/102, 42.2%). In the studied population, the distribution of BRD lower respiratory tract macroscopic lesions was not significantly related to the year, season, age, or breed-type (*p* > 0.05) (Table 2).

### 3.2. Infectious Primary Pulmonary (IPP) Macroscopic Lesions as Main Category of BRD Lower Respiratory Tract Macroscopic Lesions

Among the 115 BRD lower respiratory tract macroscopic lesions, the main category of lesions was IPP lesions (49/115, 42.6%) closely followed by TEP lesions (46/115, 40.0%). Other categories of lesions included aspiration pneumonia (11/115, 9.6%), verminous pneumonia (5/115, 4.3%), and local extension of an extra-pulmonary inflammatory process (4/115, 3.5%). The distribution of IPP macroscopic lesions was not significantly related to year, season, age or breed-type (*p* > 0.05) (Table 2).

### 3.3. FHN Bronchopneumonia as the Main Macroscopic Pattern among the IPP Macroscopic Lesions

Macroscopically, FHN bronchopneumonia was characterized by a marked to severe fibrinous pleuritis, a dark red color of parenchyma, a sharp demarcation between lesional and non-lesional tissue, a marked to severe increased consistency and the presence of marked emphysema. It was the most frequently recorded macroscopic pattern in IPP lesions (38/49, 77.6%), far ahead of suppurative bronchopneumonia (4/49, 8.2%), mixed suppurative bronchopneumonia and broncho-interstitial pneumonia (4/49, 8.2%), and broncho-interstitial pneumonia (3/49, 6%)

Macroscopic lesions of FHN bronchopneumonia were more frequently encountered during the autumn/winter season (*p* = 0.03) (Table 2).

### 3.4. FHN Bronchopneumonia as the Main Histological Pattern among the IPP Macroscopic Lesions

Fifty-three percent of IPP macroscopic lesions (26/49) were sampled for histology. FHN bronchopneumonia was also the most frequently reported histological pattern in IPP lesions (19/26, 73.1%), far ahead of suppurative bronchopneumonia (3/26, 11.5%), association of suppurative pneumonia and broncho-interstitial pneumonia (2/26, 7.7%) and broncho-interstitial pneumonia (2/26, 7.7%). It was characterized by fibrinous pleuritis, luminal exudate composed of neutrophils, fibrinous alveolitis, “oat cells”, necrotic foci and blood vessel or lymphatic thrombosis. The distribution of the FHN histological pattern was not significantly related to year, season, age or breed-type (*p* > 0.05) (Table 2).

### 3.5. Pathogen Detection

Among the 26 lungs with IPP macroscopic lesions sampled for histology and PCR, *M. haemolytica* (69.2%) and *P. multocida* (61.5%) were the most frequently detected pathogens (Table 3). *H. somni* (15.4%) and *M. bovis* (11.5%) were less frequently detected. BCoV (30.8%) was the most frequently detected virus. BRSV was infrequently detected, and BPIV-3 was not detected.

Combinations of pathogens were also described (Table 4). In cases where *M. haemolytica* was detected, it was alone in 38.9% of cases and frequently associated with *P. multocida* (50%) and with BCoV. Viruses and *M. bovis* were never detected alone.

Among the 19 lungs with a FHN-bronchopneumonia histological pattern, *M. haemolytica* was by far the most frequently detected pathogen, ahead of *P. multocida* (94.7% and 52.6% respectively). The prevalence of *H. somni* and *M. bovis* was similar to those from lungs with IPP macroscopic lesions. Bovine coronavirus was the most frequently detected virus, whereas other viruses were infrequently detected.

Detection of *M. haemolytica* was significantly (*p* < 0.001) more frequent among lungs with a histological pattern of FHN-bronchopneumonia, compared to other histological patterns. *P. multocida*, *H. somni*, *M. bovis* and viruses were not significantly more detected among lungs with a FHN-bronchopneumonia histological pattern, compared to others (data not shown).

## 4. Discussion

This study, conducted on a large data set of adult cattle necropsies, both described causes of death and investigated the macroscopic and histological patterns and pathogens associated with BRD. This study highlights that BRD is the second leading cause of death after digestive disorders and that FHN bronchopneumonia associated with *M. haemolytica* is a frequent macroscopic and histological diagnosis in adult cattle.

We described data from adult cattle that were submitted to the PULA for necropsy from January 2013 to December 2019. The sample was large, but only cattle from West France were included in the study, so we do not expect to provide a generalizable assessment of causes of death at the national level. Furthermore, the sample, consisting of both cattle that were euthanized and cattle that died unassisted, was biased toward a certain profile of diseased animals and causes of death. Cases preceded by obvious clinical signs were certainly under-represented, whereas the reverse probably occurred for sudden death and disease outbreaks. It is indeed well known that cattle necropsy submission is influenced by the number of sick and/or dead animals on the farm [13]. Consequently, farmers are more willing to pay for necropsy when faced with an outbreak of diseases. Furthermore, in the case of sudden death, adult cattle necropsy is the sole examination capable of providing a diagnosis [13].

Our first main result is that BRD was the second leading cause of death after digestive disorders. We have defined categories of causes of death based on a veterinary pathology reference book [12]. Similar to a Swiss study using the same categorization [10], we found that digestive disorders were the most frequent causes of death and that respiratory disorders were significant causes of death in adult cattle. However, a thorough comparison is difficult. For example, the classification of hardware disease associated with peritonitis, a main cause of death in adult cattle (27.8% of digestive diseases in our sample), is not straightforward. It may be classified (i) in the respiratory disorders category in studies focusing on proximate causes of death (pleuritis associated with hardware disease) [8,9,14], or (ii) in the serous membrane category if the focus is on the affected organ systems (because the main lesion is peritonitis) [10]. We have classified them in the digestive disorders category, according to Uzal et al. [12].

We can also note that a rather high proportion (13.8%) of cattle died of undetermined causes and almost half of them did not exhibit macroscopic lesions. This proportion varies from less than 5% [8,9,14] to 24–30% [15,16], depending on the studies. A more complete clinical history could decrease the proportion of undetermined causes through relevant post-mortem ancillary tests. Several authors have pointed out the need for reliable clinical histories before implementing a necropsy [10,16].

Our second main result is the large proportion of TEP lesions and IPP lesions among BRD macroscopic lesions in adult cattle. In the first place are TEP lesions, a well-known and well described affliction [17,18]. They are characterized macroscopically by multiple red to beige nodular lesions centered on the blood vessels and disseminated throughout the lung parenchyma. They are sometimes associated with pulmonary infarction [19]. TEP lesions are considered more frequent in adult than young cattle [1,17]. Therefore, it is not surprising that these lesions were frequently observed in our sample. TEP lesions are linked to hematogenous bacterial dissemination from an identified primary focus (mastitis, metritis, peritonitis, valvular endocarditis, hepatic abscess with thrombosis of the caudal vena cave, thrombosis of uterine and pelvic veins, thrombi in deep veins of the legs in recumbent (downer) cows, etc.) [19]. TEP lesions were not submitted to further investigations because TEP is a sporadic condition involving non-specific bacteria and other pyogenic bacteria, mainly *Trueperella pyogenes* [20].

In the second place, the high proportion of IPP lesions is a more remarkable result. In young cattle, IPP lesions are a significant part of BRD lesions because they are the ones observed in major collective pulmonary diseases, such as enzootic pneumonia of dairy calves or shipping fever [2,3,4,6,7]. To our knowledge, there are no published data mentioning a high proportion of IPP lesions in adult beef or dairy cattle, and information concerning the etiology of BRD in adult cattle in the literature is scarce [21]. Among IPP lesions, the most frequent macroscopic and histological pattern in our study was FHN bronchopneumonia.

Thirdly, our study shows the strong contribution of *M. haemolytica* in FHN bronchopneumonia lesions. We performed a rather complete screening of potential pathogens by using PCR that target the main pathogens involved in BRD [2,3,4,6,22,23]. However, three major pathogens were not sought: (i) *Mycobacterium bovis,* because no caseo-necrotic bronchopneumonia lesion was observed in our study—however, bovine tuberculosis infection remains at a low but persistent level in French cattle herds [24], and thus, we cannot totally exclude the possibility of having missed cases of *Mycobacterium bovis* in animals without case-necrotic lesions; (ii) bovine viral diarrhea virus; and (iii) bovine herpesvirus type 1, because control programs currently keep them at very low incidence levels in West France [25,26]. Furthermore, in West France herds are monitored annually for IBR and BVD; herds included in the study were considered free of these pathogens. In any case, *Mycobacterium bovis*, IBR and BVD are known important pathogens in respiratory diseases, and our results should be extrapolated to other areas with caution.

We used a PCR-based diagnostic approach. PCR techniques have shown a higher detection rate than conventional standard procedures for the targeted pathogens. Indeed, the isolation of these pathogens can be negatively influenced by the presence of antibiotics in the tissues administered in the terminal stage of pneumonia or by the growth of less fastidious bacteria present in the cultures [27,28]. However, the focus on PCR-based diagnostic approaches has ruled out non-targeted bacteria, particularly *Trueperella pyogenes*, a well-known agent involved in terminal stage of adult bovine pneumonia, and *Bibersteinia trehalosi,* a potentially emerging agent involved in the mortality of adult cattle [27,29,30].

PCR was performed at necropsy, i.e., at the end of the disease process, when the initiating pathogens may no longer be present in the lungs. In calf pneumonia, for instance, it is commonly accepted that viruses and mycoplasmas are the primary pathogens and that bacteria cause a secondary infection [6,19,21]. Therefore, we cannot rule out the possibility that an extensive FHN bronchopneumonia lesion causing the death of the animal prevented other lesions (viral interstitial pneumonia for instance) from being seen at the macroscopic or histological examination [31]. Further studies focusing on the early stage of pneumonia in adult cattle are needed to determine the actual involvement of viruses and mycoplasmas in adult cattle pneumonia. In the present study, the high prevalence of bovine coronavirus (8/26) observed is striking. This prevalence appears to be much higher than that of the other viruses, which were hardly detected at all. There are no published data on the current prevalence of bovine respiratory coronavirus in either young or adult cattle in France, which should be determined as a primary concern.

However, our study demonstrated the important role of *M. haemolytica* as a cause of death (preceded or not by other initiating pathogens) of adult cattle in West France. Indeed, FHN bronchopneumonia was the main macroscopic and histological pattern among IPP lesions, and *M. haemolytica* was the most frequently detected pathogen. FHN bronchopneumonia lesions are typical of those produced by *M. haemolytica* and, to a lesser extent, by *H. somni* [6,18]. In our sample, FHN bronchopneumonia lesions were clearly associated with *M. haemolytica*. Our result is in line with recently published data and reports from Great Britain. In Europe, more precisely, in several areas of the Netherlands, *M. haemolytica*-associated diseases (acute pleuropneumonia in dairy cows and polyserositis in veal calves) were increasingly seen in necropsied animals between 2004 and 2018 [7]. Outbreaks of *M. haemolytica*-associated diseases in adult cattle were also reported in Scotland [32].

Finally, little is known of the environmental, production system, immunity and pathogen factors contributing to *M. haemolytica*-associated respiratory diseases in adult cattle. Concerning environmental factors, we observed that FHN bronchopneumonia lesions were more frequent during the cold season as already mentioned in the Netherlands [7]. Concerning production systems, a particularity of our study is that we included dairy and beef cattle, and we showed that the prevalence of FHN bronchopneumonia was not linked to breed type. Further studies concerning the potential genetic or virulence particularities of *M. haemolytica* strains affecting adult cattle and immunity are needed [33,34,35,36].

## 5. Conclusions

We conducted a necropsy study on causes of death in adult cattle in West France. BRD was the second highest cause of death after digestive disorders. IPP and TEP lesions were the main categories of BRD lower respiratory tract lesions. Among IPP lesions, FHN bronchopneumonia was the main lesion and *M. haemolytica* was the most frequently detected pathogen. Our study strongly suggests that FHN bronchopneumonia lesions associated with *M. haemolytica* should be included in the differential diagnosis of adult cattle outbreaks of rapid or sudden death in West France.

## Figures and Tables

**Table 1:** List of the 8 categories used to classify the principal cause of death in necropsied adult cattle with illustrating examples.

Category	Examples of Diagnoses
1—Cardiovascular disorders	Pericarditis, endocarditis, venous thrombosis, purpura, cardiac malformations, myocardial abscessation, vascular rupture
2—Digestive disorders	Peritonitis, esophagitis, ruminal acidosis, bloating, abomasal displacement, abomasal perforating ulcer, abomasitis, hemorrhagic bowel syndrome, volvulus, intestinal herniation, intussusception, intestinal rupture, enteritis, typhlocolitis, internal parasites, hepatic diseases
3—Musculoskeletal disorders	Arthritis, fracture, dislocation, osteomyelitis, osteochondrosis, myositis
4—Reproductive disorders	Dystocia, mastitis, metritis, Caesarean operation complications, uterus prolapse, uterus hemorrhages, calving hemorrhages, uterus rupture, uterus torsion
5—Respiratory disorders	Infectious pneumonia, aspiration pneumonia, thromboembolic pneumonia
6—Systemic disorders	Septicemia, cachexia, anaphylactic shock
7—Others	Intoxication (Plant poisoning, mycotoxins, chemical poisoning)
	Dermatologic disorders (besnoitiosis, cutaneous abscessation, massive external parasites)
	Metabolic disorders (fat cow syndrome, ketosis, downer cow syndrome)
	Neurologic disorders (encephalitis, meningitis)
	Urinary disorders (pyelonephritis, nephritis)
8—Undetermined	

**Table 2:** Distribution of bovine respiratory disease (BRD) lower respiratory tract lesions; infectious primary pulmonary (IPP) lesions; fibrinous, hemorrhagic and/or necrotic bronchopneumonia (FHN BP) lesions in 737 adult cattle, according to years of necropsy, season, age and breed type (AW: Autumn/Winter, SS: Spring/Summer).

Characteristics of Necropsied Cattle		BRD Lower Respiratory Tract Macroscopic Lesions		IPP Macroscopic Lesions		FHN BP Lesions			
						Macroscopic Lesion		Histological Lesion	
		*n*	*p*	*n*	*p*	*n*	*p*	*n*	*p*
***n***		115/737		49/115		38/49		19/26	
**Year of death**	(2013–2015)	45/259	0.10	15/45	0.27	11/15	0.75	2/2	0.82
	(2016–2017)	25/222		12/25		9/12		7/9	
	(2018–2019)	45/256		22/45		18/22		10/15	
**Season of death**	AW	76/445	0.21	32/76	1	28/32	0.03	15/18	0.15
	SS	39/292		17/39		10/17		4/8	
**Age of death**	(2–3)	34/242	0.65	15/34	0.96	10/15	0.48	3/7	0.11
	(4–5)	39/249		16/39		13/16		9/10	
	>5	42/246		18/42		15/18		7/9	
**Breed type**	Dairy	95/557	0.07	42/95	0.61	32/64	1	16/22	1
	Beef	20/180		7/20		6/12		3/4	

**Table 3:** Frequency of PCR-detected pathogens in the lungs of adult cattle with infectious primary pulmonary (IPP) macroscopic lesions and with histological pattern of fibrinous, hemorrhagic and/or necrotic bronchopneumonia (FHN BP).

Pathogens	Frequency of Positive PCR (%) for IPP Macroscopic Lesions (*n* = 26)	Frequency of Positive PCR (%) for Histological Pattern of FHN-BP (*n* = 19)
Bacteria		
*Mannheimia haemolytica*	69.2 (18)	94.7 (18)
*Pasteurella multocida*	61.5 (16)	52.6 (10)
*Histophilus somni*	15.4 (4)	15.8 (3)
*Mycoplasma bovis*	11.5 (3)	15.8 (3)
Viruses		
Bovine coronavirus	30.8 (8)	36.8 (7)
Bovine respiratory syncytial virus	3.8 (1)	5.3 (1)
Parainfluenza type-3 virus	0 (0)	0 (0)

**Table 4:** Frequency of co-infections between pathogens (*n* = 26).

Pathogens		Co-Infections	
	Frequency		Frequency
*Mannheimia haemolytica*	18/26	*M.h.* alone	7/18
		*M.h.* + *P.m.*	4/18
		*M.h.* + *P.m.* + other pathogen	5/18
		*M.h.* + other pathogens	2/18
*Pasteurella multocida*	16/26	*P.m.* alone	4/16
		*P.m.* + M.h.	4/16
		*P.m.* + *M.h.* + other pathogens	5/16
		*P.m.* + other pathogens	3/16
*Histophilus somni*	4/26	*H.s.* alone	0/4
		*H.s.* + *P.m.*	1/4
		*H.s.* + *P.m.* + BCoV	1/4
		*H.s.* + *P.m.* + *M.h.* + *M.b.* + BCoV	1/4
		*H.s.* + *M.h.* + *M.b.* + BCoV	1/4
*Mycoplasma bovis*	3/26	*M.b.* alone	0/3
		*M.b.* + *P.m.* + *M.h.*	1/3
		*M.b.* + *H.s.* + BCoV	1/3
		*M.b.* + *P.m.* + *H.s.* + BCoV	1/3
BCoV	8/26	BCoV alone	0/8
		BCoV + *M.h.* + *P.m.*	3/8
		BCoV + *M.h.*	1/8
		BCoV + *P.m.*	1/8
		BCoV + *M.h.* + others	2/8
		BCoV + others	1/8
BRSV	1/26	BRSV alone	0/1
		BRSV + *P.m.* + *M.h.* + BCoV	1/1
BPIV-3	0/26	/	/

*M.h.: Mannheimia haemolytica, P.m.: Pasteurella multocida, H.s.: Histophilus somni, M.b.: Mycoplasma bovis*, BCoV: bovine coronavirus, BRSV: bovine respiratory syncytial virus, BPIV-3: bovine parainfluenza type-3 virus.

## Data Availability

The data presented in this study are available on request from the corresponding author. The data are not publicly available due to privacy restrictions.
